# Household Hints and Recipes

**Published:** 1878-07

**Authors:** 


### Household Hints & Recipes.

TABLE-SALT IN MILK FOR CHILDREN.—Dr. J. Q. Smith says that, when cow's milk disagrees with young children, the addition of a small quantity of table-salt will often correct the difficulty.

TO PROMOTE THE GROWTH OF WHISKERS.—

| | |
|---|---|
| Veal fat | 1 pound. |
| Tincture of cantharides | 1 ounce. |
| Oil of mace | ½ ounce. |
| Oil of petit-grain | 30 drops. |

TEA HAIR TONIC.—

| | |
|---|---|
| Strong infusion of black tea, | 1 pint. |
| Bay rum, | 4 ounces. |
| Oil of lavender, | 1 drachm. |
| Alcohol, | 4 ounces. |
| Glycerine, | 4 " |

LEMON CORDIAL.—

| | |
|---|---|
| Fresh lemon peel, | 2 ounces. |
| " orange " | 1 ounce. |
| Dry lemon peel, | 2 ounces. |
| Diluted alcohol, | 1 gallon. |
| Water and syrup, of each, | 6 pints. |

LIQUID SHOE-VARNISH.—You may try the following: Dissolve 8 ozs. of shellac, and ½ oz. of camphor, in 2 pints of alcohol; when dissolved, add 1 ounce of lamp-black. Shake the mixture before using, and apply it with a small paint brush. The addition of about 1 oz. of gum sandarac is said to improve it.

TO CLEAN PAINT.—An ounce of pulverized borax, a pound of best brown soap in small pieces and three quarts of water are to be mixed and put on the fire. It should simmer until the soap dissolves, being frequently stirred. Do not allow it to boil. Use with flannel and rinse off as soon as the paint is clean. This mixture is also recommended for washing clothes.

TO PRESERVE FRESH FRUITS.—Mr. Antonio dal Piaz recommends to place the fresh, sound fruits into a solution of sugar, made by dissolving in 1 litre (about 34 fl. oz.) of water 100-500 grammes (3½ to 17½ oz.) of sugar—the quantity varies with the acidity of the fruit—and 2½ to 3 grammes (40 to 50 grains) of saliyclic acid. Cherries, currants, blackberries, pears, grapes, gooseberries, etc., may thus be preserved, even in loosely covered vessels, without injuring the natural aroma.

TEST FOR MILK.—The following test, given by the *Michigan Medical News*, for watered milk is said to be reliable, and certainly its simplicity commends it: Dip a well polished knitting needle into the suspected fluid and withdraw it immediately in a perpendicular position. Should the sample contain no water, some of the fluid will cling to the needle; should water be present, however, even in a very small quantity, this adhesiveness will be lost and the needle will be withdrawn clear.

DETECTION OF ARTIFICIAL BUTTER.—A simple and ingenious process, recommended by H. Hager in *Phar. Centralhalle*, consists in melting the butter at a gentle heat, allowing it to become quite clear by standing, and saturating a cotton lamp wick with the limpid fat. The wick is then ignited, and extinguished after about two minutes. If the butter is genuine, the odor will be that of strongly fried butter, while the presence of oleomargarine is betrayed by the well-known unpleasant odor of a tallow candle recently extinguished.

USES OF THE LEMON.—The London *Lancet* says: "Few people know the value of lemon-juice. A piece of lemon bound upon a corn will cure it in a few days; it should be renewed night and morning. A free use of lemon-juice and sugar will always relieve a cough. Most people feel poorly in the spring, but if they would eat a lemon before breakfast every day for a week—with or without sugar, as they like—they would find it better than any medicine. Lemon-juice, used according to this recipe, will sometimes cure consumption: Put a dozen lemons into cold water and slowly bring to a boil; boil slowly until the lemons are soft, then squeeze until all the juice is extracted; add sugar to your taste and drink. In this way use one dozen lemons a day. If they cause pain, lessen the quantity, and use only five or six a day until you are better, and then begin with a dozen a day. After using five or six dozen the patient will begin to gain flesh and enjoy food. Hold on to the lemons, and still use them very freely for several weeks more. Another use for lemons is for a refreshing drink in summer, or in sickness at any time. Prepare as directed above and add water and sugar. But in order to have this keep well, after boiling the lemons, squeeze and strain carefully; then to every half pint of juice add one pound of loaf or crushed sugar, boil and stir a few minutes more until the sugar is dissolved, skim carefully and bottle. You will get more juice from the lemons by boiling them, and the preparation keeps better."

BLACK INK.—The most agreeable bluish-black writing ink, which never gets mouldy, does not corrode steel pens, and may be prepared by anybody without trouble and at a trifling expense only, is made by dissolving 1 part of nigrosine in 80 parts of water. One pound of nigrosine may be bought for about $3.50.

---

ARTIFICIAL FLOWER BAROMETERS.—Artificial flowers, called barometers, are now being exhibited in a number of Parisian opticians' shops. They are colored with a material composed of chloride of cobalt. When exposed to sun and dry air, the leaves become deep blue; when the air is saturated with moisture they become pinky. All the intermediate shades are easily observed.—*London Journal of Horticulture.*

---

GLUE FOR DAMP ATMOSPHERES.—A glue, fast in a damp atmosphere, for fastening labels on bottles, etc., may be made by macerating 5 parts of good glue in 20 parts of water, for a day, and then adding to the liquid 9 parts of sugar-candy and 3 parts of gum arabic. The mixture can be brushed upon paper while luke-warm: it keeps well, does not stick together, and when moistened adheres firmly.

---

TO MEND INDIA-RUBBER.—The correspondent wishes a preparation which will mend India-rubber goods, either hard or soft. Has tried among many others a solution of crude rubber and rosin in carbon disulphide, but finds it to be of little use.

We doubt whether there is a preparation which will cement together hard or soft rubber equally well. Unvulcanized rubber may be mended or cemented by using the following preparation: Take 16 parts gutta-percha, 4 parts India rubber, 2 parts common pitch, and one part linseed oil. Melt them together, and use it hot. We have never tried this on hard rubber, but think it may possibly answer. Equal parts of asphaltum and India-rubber melted together might also answer.

---

PREVENTIVE OF LEAD POISONING.—Workmen employed in the manufacture of white lead are always liable to lead poisoning, both by inhaling the dust and in touching the lead with the hands. Various correctives for this have been employed, and among these the latest and most simple (proposed in the *American Manufacturer*), is a careful washing of the hands in petroleum. Three washings a day are reported to be sufficient to prevent all serious danger by poisoning. The benzole in the petroleum is said to scour the skin and remove the dust of lead, and the fatty substance in the oil prevents the absorption of the lead salts. The experiment made in petroleum used in this manner gives such good results that it is proposed to use the same material as a guard against poisoning in other industries where the salts of copper or mercury are employed.

---

TO POLISH SILVER.—Have a dish of very hot water at hand; a soft linen rag for washing; also a very soft cloth of good size for drying; a cake of silver soap; rub soap on your washing-cloth, and rub separately each piece of silver, using a fine quality of silver or tooth brush, especially on the ornamental work, about the handles, and wherever necessary; dry at once, and polish vigorously with a chamois skin; this should be done once a week, and better still twice, and there will be no use for powder, which in time will unavoidably turn the silver dark.

---

GREEN INK.—Green aniline (soluble), 2 drachms; alcohol, 14 ounces; glycerin, 2 ounces; mucilage of gum-arabic, 4 drachms; dissolve the aniline in the alcohol and add the other ingredients [NELSON]. In this case most of the mucilage separates again on standing; nevertheless, it appears to improve the body decidedly.

*Klaproth's Green Ink.*—Crystallized copper acetate (verdigris), 4; cream of tartar, 2; water, 16 parts. Boil the two salts in a glass or clean copper vessel with the water, until the solution has acquired an intense green color. Then filter, add 1 part of mucilage, and bottle.

*Green Chrome Ink.*—Potassium bichromate, 10; hydrochloric acid, 10; alcohol, 10; gum-arabic, 10; water, 30 parts. The finely powdered bichromate contained in a *capacious* porcelain or stone vessel is covered with the hydrochloric acid. After standing an hour, the alcohol is very gradually and in small quantities added. The liquid must be constantly stirred with a glass-rod, and after each addition of alcohol, the effervescence must be allowed to subside before adding more. To the green liquid thus produced powdered sodium carbonate is added, as long as effervescence takes place. As soon as a permanent green precipitate begins to form, the addition of soda is interrupted, the vessel covered and set aside for a week, the contents then transferred to a filter, to separate the solid salt formed, and the filtrate diluted with sufficient water to give it the proper shade of color. Finally the gum-arabic is dissolve in the liquid. This ink penetrates the paper very energetically, and is almost indestructible.

